# US Parents’ Acceptance of Learning About Mindfulness Practices for Parents and Children: National Cross-sectional Survey

**DOI:** 10.2196/30242

**Published:** 2021-11-02

**Authors:** Mala Mathur, Bradley R Kerr, Jessica C Babal, Jens C Eickhoff, Ryan J Coller, Megan A Moreno

**Affiliations:** 1 Department of Pediatrics School of Medicine and Public Health University of Wisconsin-Madison Madison, WI United States; 2 Department of Biostatistics and Medical Informatics School of Medicine and Public Health University of Wisconsin-Madison Madison, WI United States

**Keywords:** mindfulness, mental health, general pediatrics, pediatrics, children, parents, acceptability, well-being, parenting

## Abstract

**Background:**

Mindfulness practices are associated with improved health and well-being for children. Few studies have assessed parents’ acceptance of learning about mindfulness practices.

**Objective:**

This study aims to assess parents’ beliefs and interest in learning about mindfulness, including from their health care provider, and differences across demographic backgrounds.

**Methods:**

We conducted a national cross-sectional survey of parents with children aged 0-18 years in October 2018. Measures included beliefs and interest in learning about mindfulness. These measures were compared across demographic backgrounds using chi-square analysis. Multivariate linear and logistic regression analyses were used to perform adjusted comparisons between demographic backgrounds.

**Results:**

Participants (N=3000) were 87% (n=2621) female and 82.5% (n=2466) Caucasian. Most (n=1913, 64.2%) reported beliefs that mindfulness can be beneficial when parenting, 56.4% (n=1595) showed interest in learning about mindfulness to help their child stay healthy, and 40.8% (n=1214) reported interest in learning about mindfulness from their health care provider. Parents with a college degree 49.6% (n=444) were more likely to report interest in learning about mindfulness from a health care provider compared to those without 37.1% (n=768; *P*<.001). Parents interested in learning about mindfulness were more likely to be male 62.6% (n=223; *P*<.001). There was no significant difference in interest in learning about mindfulness from a health care provider based on race.

**Conclusions:**

This study indicates that many parents believe mindfulness can be beneficial while parenting and are interested in learning how mindfulness could help their child stay healthy. Findings suggest there is an opportunity to educate families about mindfulness practices.

## Introduction

Anxiety and depression affect an estimated 1 in 20, or 2.6 million, children in the United States [1]. The high prevalence of these mental health conditions in our nation’s youth adversely impacts overall physical health, school attendance and achievement, alcohol and drug use, family discord, violence, suicide, and health care costs [2,3]. It is imperative for health care systems to use a variety of approaches to the prevention and treatment for these and other mental health conditions [4].

Mindfulness techniques represent one approach showing promise in the prevention and treatment of mental illness. Mindfulness is generally defined as “paying attention in a particular way: on purpose, in the present moment and nonjudgmentally” [5]. Formal mindfulness approaches include a variety of activities including mindful breathing, mindful walking, meditation, and yoga. Informal mindfulness practices include bringing a mindful approach to activities of everyday living, including mindful eating or mindful washing of dishes [6]. Mindfulness therapies address emotional self-regulation and are a commonly used psychological approach to reduce stress and discomfort [7].

Mindfulness interventions may benefit children directly, through their own practice, and indirectly, when their parents use this technique [8,9]. Previous studies show that mindfulness interventions for children reduce anxiety and stress [10,11]. A growing body of literature suggests mindfulness techniques practiced by parents can reduce parenting stress [12,13] and have positive mental health impacts on children [8,14-16] and on parent-child interactions [17].

Although there is increasing evidence supporting mindfulness as an approach to improve mental health, this practice has been most used by women, particularly women who identify as White and are of higher socioeconomic status [18]. However, mindfulness may be especially beneficial for socially marginalized families (ie, based on race and ethnic background), families with lower socioeconomic status, and those with limited access to mental health resources who may be at higher risk of mental health conditions [19]. Understanding the views of diverse groups toward mindfulness is an important step toward teaching these practices to improve the mental health of all children.

However, the acceptance of parents from diverse backgrounds toward learning about mindfulness independently or from their health care provider remains unknown. This exploratory study aims to understand parental acceptance of mindfulness including the prevalence of parents who believe mindfulness could be beneficial in parenting and would be interested in learning about mindfulness. Further, the study aims to understand differences in beliefs and interest in learning about mindfulness among parents across parent gender, race, ethnicity, education, and income.

## Methods

This national cross-sectional survey study was conducted in October 2018 as part of a larger study involving parents’ perspectives of pediatric health care, which was estimated to take 10 to 20 minutes to complete. The University of Wisconsin Education and Social/Behavioral Sciences Institutional Review Board deemed this study as exempt from institutional review board approval (#2018-1051).

### Participants

We recruited a national panel of parents representing all regions of the United States. Survey panels are an approach to research in which individuals sign up to be on lists to receive survey invitations. Previous studies have supported these survey panels as an effective approach with broader geographic reach than traditional survey approaches [20,21]. We selected the online survey platform Qualtrics to conduct this study. Qualtrics recruits from geographically diverse areas of the United States to generate panels of participants who are interested in receiving invitations to participate in future surveys. Upon joining Qualtrics, panelists complete demographic assessments so that survey invitations can be targeted to eligible survey populations. As participation incentive, participants receive “Qualtrics Points” for survey completion, which can be applied toward purchases such as gift cards and airline miles.

We requested that Qualtrics recruit 3000 parents. Survey invitations were sent by email to relevant panels of potentially eligible adult participants. Interested panelists then completed screening questions with eligibility criteria specific to this study: English-speaking, 18 years or older, and parent of a child younger than 18 years. Participants completed written informed consent through the online Qualtrics platform. The survey closed when the goal sample size of 3000 participants meeting eligibility criteria was reached.

### Measures

We provided a series of statements assessing parent acceptance of mindfulness that participants rated using Likert scales. To assess parental beliefs about the benefits of mindfulness, the following statement was provided: I believe mindfulness techniques can be beneficial when parenting my child/children. Statements about interest in learning more about mindfulness included: I am interested in learning about how mindfulness could lead to benefits for my child as an individual, I am interested in learning about how mindfulness could help my child stay healthy, I am interested in learning about how mindfulness could lead to benefits for myself as an individual and in my abilities to parent my child, and I am interested in learning about mindfulness from my health care provider.

Statements for this survey were developed by the study team. After development, these were piloted among a group of general pediatricians and parents, and modified based on their feedback. All survey items were framed as statements with which participants indicated their agreement on a 5-point Likert scale from “strongly disagree” to “strongly agree.” An option of “don’t know” was also offered.

Demographic variables included parent gender, race, ethnicity, education, and income.

### Analysis

Descriptive statistics were calculated for demographics and measures pertaining to mindfulness benefits and interest in learning about mindfulness practices. Analyses were focused on assessing proportions of participants with positive views about mindfulness practices. Thus, participants reporting mindfulness-related perceptions were categorized into three groups: (1) those indicating positive beliefs or interest in learning about mindfulness (answered “agree/strongly agree”), (2) those indicating neutral or negative beliefs or interest in learning about mindfulness, and (3) those indicating “don’t know.”

Beliefs and interest toward mindfulness were compared across demographic categories (parent gender, race, ethnicity, education, income) using chi-square analysis. Multivariate linear and logistic regression analyses were used to perform adjusted comparisons between demographic categories. Demographic characteristics (age, gender, education, income, race, ethnicity), excluding the demographic characteristic of the primary comparison, were included as covariates in the multivariate linear and logistic regression models. For example, when comparing response patterns of beliefs and interest toward mindfulness between males versus females, age, education, income, race, and ethnicity were included as covariates. All reported *P* values were 2-sided, and *P*<.05 was used to define statistical significance. Statistical analyses were conducted using SAS software (SAS Institute), version 9.4.

## Results

Our sample included 3000 participants. Among them, 87.9% (n=2621) were female, 82.5% (n=2466) were White, 88.7% (n=2645) were non-Hispanic, 69.9% (n=2093) had no college degree, and 47.2% (n=1410) had a family income less than US $50,000. All 50 US states and all four regions were represented (Table 1).

**Table 1:** Demographic characteristics of parent participants (N=3000).

		Participant, n (%)
**Gender**		
	Female	2621 (87.9)
	Male	360 (12.1)
	Other/missing	19 (0.006)
**Race**		
	White	2466 (82.5)
	Black	266 (8.9)
	Other	167 (5.6)
	Asian	90 (3.0)
	Other/missing	11 (0.003)
**Ethnicity**		
	Non-Hispanic	2645 (88.7)
	Hispanic	338 (11.3)
	Missing	17 (0.005)
**Education**		
	No college degree	2093 (69.9)
	College degree	900 (30.0)
	Missing	7 (0.002)
**Income (US $)**		
	<20,000	400 (13.4)
	20,000-34,999	533 (17.8)
	35,000-49,999	477 (16.0)
	50,000-74,999	494 (16.5)
	75,000-99,999	362 (12.1)
	100,000-149,999	320 (10.7)
	150,000-199,999	168 (5.6)
	≥200,000	122 (4.1)
	Prefer not to say	114 (3.8)

### Belief That Mindfulness Can Be Beneficial in Parenting

In total, 64.2% (n=1913) of the 3000 participants agreed that mindfulness can be beneficial when parenting, 30.2% (n=906) of participants reported they disagreed or were neutral, and 5.3% (n=159) of parents stated they “don’t know.” Multivariate analysis showed that those with a college degree were more likely to believe that mindfulness can be beneficial when parenting compared to those without a college degree (*P*<.001) when adjusting for age, gender, and ethnicity. There was no significant difference in the belief that mindfulness can be beneficial while parenting based on parent gender. Parents who reported an income of less than US $20,000 were less likely to report a belief that mindfulness can be beneficial compared to those who reported earning US $50,000-$75,000 (*P*=.004); US $100,000-$149,999 (*P*=.01); US $150,000-$199,999 (*P*=.02); and over US $200,000 (*P*=.004; see Multimedia Appendix 1 for all findings on parent beliefs about mindfulness).

### Interest in Learning About Mindfulness

Among all 3000 participants, 53.1% (n=1581) reported they agreed that they were interested in learning how mindfulness can lead to benefits for their child, while 42.2% (n=1259) reported they disagreed or were neutral and 4.5% (n=135) answered “don’t know.” Over half of participants (n=1595, 53.7%) reported they agreed they were interested in learning about how mindfulness could help their child stay healthy, while 41.5% (n=1232) disagreed or were neutral and 4.9% (n=145) answered “don’t know.” About half of participants (n=1499, 50.4%) responded that they were interested in learning about how mindfulness could lead to benefits for themselves and their abilities to parent their child, while 44.8% (n=1330) disagreed or reported they were neutral to this statement and 5.3% (n=159) answered “don’t know.” Overall, 40.8% (n=1214) of participants reported interest in receiving information about mindfulness from their health care provider, while 54.5% (n=1621) reported they disagreed or were neutral and 4.7% (n=139) answered “don’t know.”

Parents with a college degree (n=444, 49.6%) were more likely to report interest in learning about mindfulness from their health care provider than those without a college degree (n=768, 37.1%; *P*<.001). Parents interested in learning about mindfulness from their health care provider were also more likely to identify as male (n=223, 62.6%) than female (n=987, 38.0%; *P*<.001). A total of 51.1% (n=46) of Asian, 41.0% (n=1003) of White, and 39.3% (n=104) of Black parents reported interest in receiving information about mindfulness from their health care provider; these differences were not significant. Parents who earned less than US $20,000 were less likely to report interest in learning about mindfulness from a health care provider than those reporting US $35,000-$49,999 (*P*=.03); US $100,000-$149,999 (*P*=.045); and those who earned over US $200,000 (*P*=.005; see Multimedia Appendices 1 and 2).

## Discussion

This exploratory study provides insight into parents’ beliefs and interest toward learning about mindfulness. Over half of parents reported believing that mindfulness can be beneficial while parenting and indicated interest in learning more about how mindfulness could keep their child healthy. Males and college-educated parents were more likely to report that they were interested in learning about mindfulness from their health care provider. Our study did not find differences in interest in receiving information about mindfulness from parents’ health care provider based on race but did find that some higher income groups were more likely to show interest in learning about mindfulness than those making less than US $20,000 a year.

Our findings suggest that most parents (n=1913, 64.2%) believe mindfulness can be beneficial while parenting and are interested in learning about mindfulness to benefit their child, but some parents (n=1259, 42.3%) may not be interested in learning about how mindfulness could benefit their child. A possible reason these parents did not show interest in learning about mindfulness is that some may believe mindfulness includes only formal practices, which take time, without realizing that informal mindfulness practices can be incorporated easily into their day. Some may also have had previous experience with practicing mindfulness and may not desire any further education. Similar to previous research, these findings highlight that adults may have different levels of readiness to learn about and engage with mindfulness practices [18]. For some parents, more education may be needed to inform parents of the benefits. For parents who may have tried formal mindfulness techniques and not continued the practice, an understanding of their experiences is needed. Additional research is needed to develop strategies to educate and engage families about mindfulness practices both formal and informal.

Although many parents reported interest in learning about mindfulness, less than half were interested in learning about mindfulness from their health care provider. There may be several possible reasons for this finding. It is possible many parents think of health care providers as focused mainly on physical health and do not perceive their health care provider as a knowledgeable source of information about mindfulness. Furthermore, parents may not perceive the busy health care provider’s office as a desired setting to learn about mindfulness and may prefer to learn about it through another venue. There are increasing numbers of online resources that offer mindfulness and meditation practices, which could potentially benefit children and families. Sharing these digital resources (web sites and apps for smartphones) with families may increase accessibility by reducing the barriers of cost and transportation. Although digital resources offer one option, more consideration needs to be given to how parents can access information about mindfulness training. For those parents who are interested in receiving information from their health care provider, future studies should explore preferences in how they prefer to learn about mindfulness in a health care setting.

This study indicated that males were more likely to be interested in receiving information about mindfulness from their health care provider compared to females. This contrasts with a recent (2017) national survey in which more women reported using yoga and meditation in the past 12 months compared to men [22]. The findings from this study that men reported more interest in learning about mindfulness is especially important given the positive impact that fathers’ mental health can have on child health outcomes from infancy to adolescence and the increasing contribution that fathers play in caring for their children [23]. A recent meta-analysis of fathers’ mental health showed that paternal depression was correlated with child and adolescent internalizing symptoms [24], which suggests the importance of supporting paternal mental health to positively impact children’s mental health. Given the impact of the paternal mental health on children, and fathers’ interest to learn about mindfulness, mindfulness education may be an important tool for supporting fathers in caring for their children.

This study did not find evidence that parents’ interest in learning about mindfulness from their health care provider differed across racial background. In contrast, previous work has suggested that Black populations engage less frequently with mindfulness than White populations [18]. It is possible this difference is due to racial bias resulting from health care providers assuming that non-White parents lack interest in mindfulness practice. Examined critically, it may be that historically less frequent engagement in mindfulness may reflect a lack of referral from health care providers (unconscious bias). More research is needed to understand the reasons why non-White families may engage less in mindfulness practices when their interest in learning about the practice may not differ.

The study also found differences in participants’ beliefs and interest in learning about mindfulness from their health care provider based on income. Those families who earned less than US $20,000 per year (approximately equivalent to the US poverty level for a family of 3 people) [25] were less likely to believe mindfulness could be beneficial and less likely than other income groups to be interested in learning about mindfulness from their health care provider. It is possible that parents living below the poverty line may not have access to health care, and this may affect their interest in learning about mindfulness from a health care provider. This finding is important since studies suggest that people with low incomes have risks that correlate with higher diagnoses of mental illness [26] and might benefit from mindfulness practices more than in other income groups. Addressing mental health issues with mindfulness practices, both formal and informal, may offer an additional resource to support the mental health of parents living in poverty. However, additional work is needed to explore approaches of providing access to mindfulness resources for these families.

Our study has limitations to consider. First, there were demographic differences between our sample and representation in the United States. For example, over 87% of participants identified as female. Further, about 8% of participants identified as Black, while in the United States, those identifying as Black make up 13% of the population [27]. Similarly, in our study, individuals identifying as Hispanic represented about one-tenth of the sample compared to over 18% of the US population [27]. Second, this study did not include parents who were non–English-speaking, while those who speak a language other than English at home comprise more than one-fifth of the US population [27]. Future studies investigating the perspectives of these populations would be of the utmost importance to capture a more representative sample of families in the United States. Finally, parents who chose to participate in this survey through the Qualtrics platform all had access to the internet, and perspectives of those without internet access may not be represented.

This study indicates that a majority of parents believe mindfulness can be beneficial while parenting, and many parents are interested in learning how mindfulness could help their child stay heathy. With the growing body of literature showing associations between mindfulness practice and mental wellness, further research should examine the perceptions and experiences of those who do not consider mindfulness beneficial. For the parents who are interested in learning more, particularly fathers, additional research is needed about how parents would like to learn about these resources. Future studies should also examine effective methods for delivering mindfulness information and resources to parents of lower household incomes including how to develop accessible mindfulness training programs.

## Appendix

Multimedia Appendix 1 Parents who believe mindfulness is beneficial when parenting.

Multimedia Appendix 2 Parents reporting interest in learning about mindfulness from their health care provider.
