# Supplementary figures and images for: US Parents’ Acceptance of Learning About Mindfulness Practices for Parents and Children: National Cross-sectional Survey
Source: JMIR Pediatr Parent. 2021 Nov 2;4(4):e30242. doi: 10.2196/30242 (PMC8596283; doi:10.2196/30242)

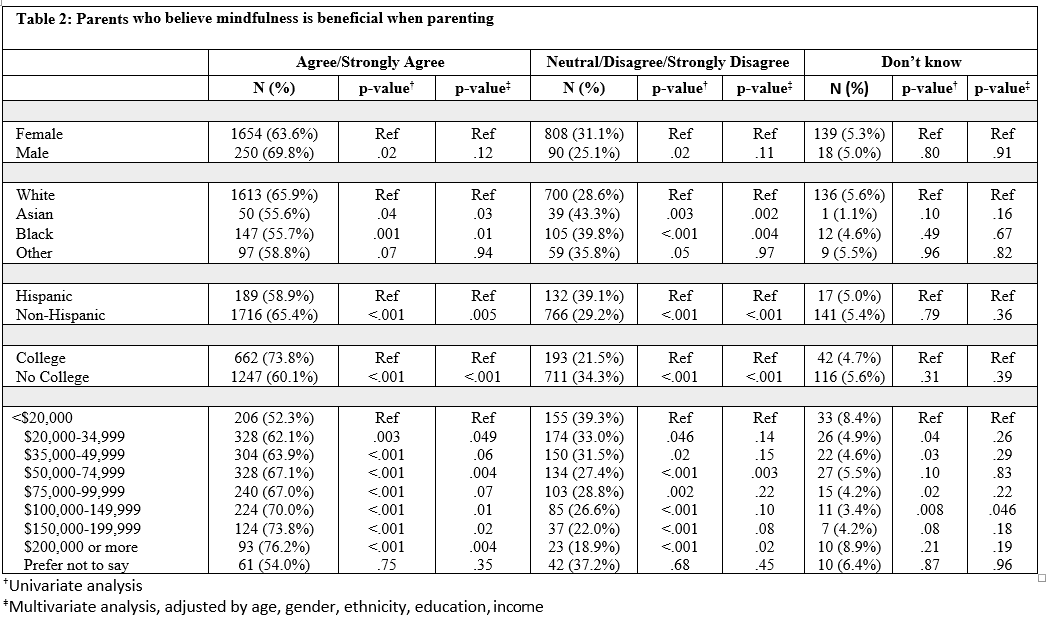

Supplement: Multimedia Appendix 1 [file pediatrics_v4i4e30242_app1.png]

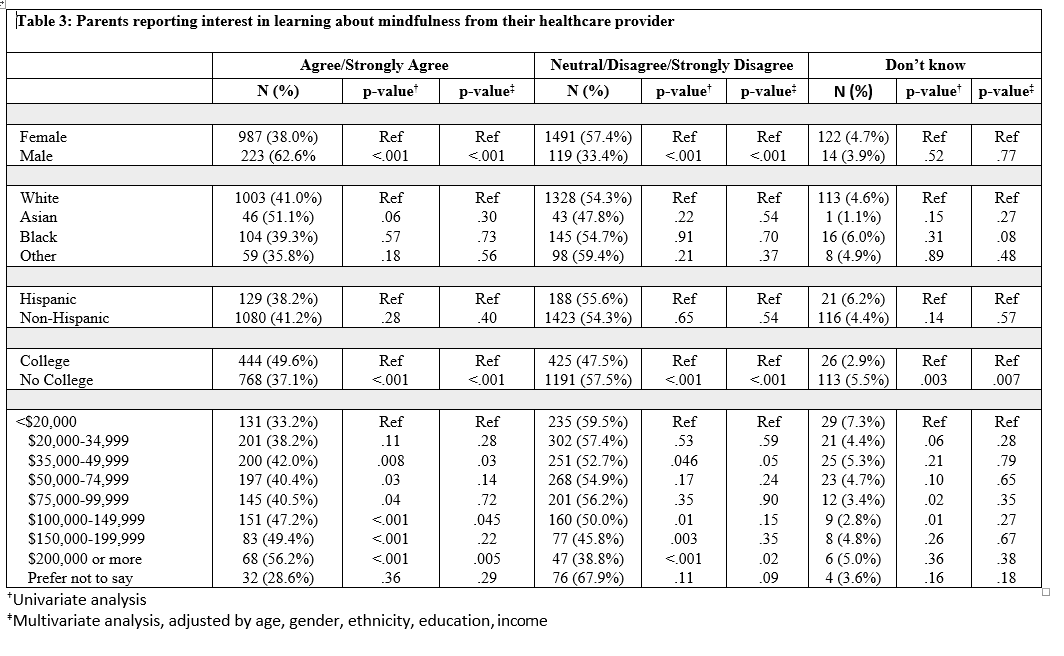

Supplement: Multimedia Appendix 2 [file pediatrics_v4i4e30242_app2.png]
